# Streptococcus *oriscaviae* sp. nov. Infection Associated with Guinea Pigs

**DOI:** 10.1128/spectrum.00014-22

**Published:** 2022-05-05

**Authors:** Jade L. L. Teng, Yuanchao Ma, Jonathan H. K. Chen, Ruibang Luo, Chuen-Hing Foo, Tsz Tuen Li, Jordan Y. H. Fong, Weiming Yao, Samson S. Y. Wong, Kitty S. C. Fung, Susanna K. P. Lau, Patrick C. Y. Woo

**Affiliations:** a Department of Microbiology, Li Ka Shing Faculty of Medicine, The University of Hong Kong, Hong Kong, China; b Department of Microbiology, Queen Mary Hospital, The University of Hong Kong, Hong Kong, China; c Department of Computer Science, The University of Hong Kong, Hong Kong, China; d Department of Pathology, United Christian Hospital, Hong Kong, China; The Pennsylvania State University

**Keywords:** *Streptococcus oriscaviae*, novel species, guinea pigs, bite wound, infection

## Abstract

Pet bite-related infections are commonly caused by the pet’s oral flora transmitted to the animal handlers through the bite wounds. In this study, we isolated a streptococcus, HKU75^T^, in pure culture from the purulent discharge collected from a guinea pig bite wound in a previously healthy young patient. HKU75^T^ was alpha-hemolytic on sheep blood agar and agglutinated with Lancefield group D and group G antisera. API 20 STREP showed that the most likely identity for HKU75^T^ was S. suis I with 85.4% confidence while Vitek 2 showed that HKU75^T^ was unidentifiable. MALDI-TOF MS identified HKU75^T^ as Streptococcus suis (score of 1.86 only). 16S rRNA gene sequencing showed that HKU75^T^ was most closely related to *S. parasuis* (98.3% nucleotide identity), whereas partial *groEL* and *rpoB* gene sequencing showed that it was most closely related to S. suis (81.8% and 89.8% nucleotide identity respectively). Whole genome sequencing and intergenomic distance determined by ANI revealed that there was <85% identity between the genome of HKU75^T^ and those of all other known Streptococcus species. Genome classification using concatenated sequences of 92 bacterial core genes showed that HKU75^T^ belonged to the Suis group. *groEL* gene sequences identical to that of HKU75^T^ could be directly amplified from the oral cavities of the two guinea pigs owned by the patient. HKU75^T^ is a novel Streptococcus species, which we propose to be named *S. oriscaviae*. The oral cavity of guinea pigs is presumably a reservoir of *S. oriscaviae*. Some of the reported S. suis strains isolated from clinical specimens may be *S. oriscaviae*.

**IMPORTANCE** We reported the discovery of a novel Streptococcus species, propose to be named Streptococcus oriscaviae, from the pus collected from a guinea pig bite wound in a healthy young patient. The bacterium was initially misidentified as S. suis/*S. parasuis* by biochemical tests, mass spectrometry. and housekeeping genes sequencing. Its novelty was confirmed by whole genome sequencing. Comparative genomic studies showed that *S. oriscaviae* belongs to the Suis group. *S. oriscaviae* sequences were detected in the oral cavities of the two guinea pigs owned by the patient, suggesting that the oral cavity of guinea pigs could be a reservoir of *S. oriscaviae*. Some of the reported S. suis strains may be *S. oriscaviae*. Further studies are warranted to refine our knowledge on this novel Streptococcus species.

## INTRODUCTION

There is an increasing number of pet-related infections worldwide (1). A significant proportion of these infections are due to the owners being accidentally bitten by their pets, leading to dog-bite infections, cat-bite infections, etc. caused by the oral flora of the pets being transmitted to the animal handlers through the bite wounds (1). In fact, some bacterial species are characteristically found in bite wounds inflicted by a specific group of animals. For instance, Pasteurella canis and Capnocytophaga canimorsus are frequently isolated in dog-bite wound infections (2, 3), as these are part of the normal flora of the canine oral cavity. In addition to dogs and cats, guinea pigs are becoming an increasingly popular choice of pet (4). Previous studies have shown that a diverse population of Streptococcus species, such as Streptococcus parasanguinis, Streptococcus mitis, and Streptococcus suis, as well as some unidentified hemolytic isolates, inhabited the oral cavities of guinea pigs (4, 5). These streptococci are potentially pathogenic for the animal host, and can cause zoonotic infections in humans.

The genus Streptococcus currently comprises more than 112 species, some of which are important human pathogens that cause significant morbidity and mortality worldwide. Traditionally, streptococci have been classified into alpha-hemolytic, beta-hemolytic, and non-hemolytic depending on the type of hemolysis that the bacterium generated on blood agar. The beta-hemolytic streptococci were further subclassified by Lancefield grouping, although some alpha-hemolytic and nonhemolytic streptococci also reacted with certain Lancefield antisera. As a result of the widespread use of PCR and DNA sequencing throughout the last 2 decades, genotypic methods like amplification and sequencing of universal gene targets represent an alternative method for classification and identification of Streptococcus. Among the various universal gene targets that have been studied, the 16S rRNA gene has been the most widely used (6, 7). However, some studies have shown that the 16S rRNA gene failed to provide sufficient resolution and to delineate Streptococcus species into proper taxonomic groupings under some circumstances (8–11).

Recently, a Gram-positive coccus, strain HKU75^T^, was isolated from the purulent discharge of a wound induced by a guinea pig bite. Although the bacterium was identified as S. suis by matrix-assisted laser desorption ionization time-of-flight mass spectrometry (MALDI-TOF MS) with a score of 1.86, the history of the patient was not compatible with a case of S. suis infection, which is often associated with a contact history of pigs. Moreover, the phenotypic characteristics of the bacterium also did not fit into patterns of any known species. A comparison based on the complete 16S rRNA gene sequences (1,557 bp) showed that there was 98.3% base identity between the 16S rRNA gene of HKU75^T^ and that of the most closely related species, *S. parasuis* SUT-286^T^, and whole genome sequencing confirmed that it is a previously undescribed bacterium. In this study, we describe the phenotypic and genotypic characterization of this novel bacterium. In addition, we also investigated the presence of it in the oral cavity of the corresponding guinea pigs. On the basis of these studies, we propose a new species, Streptococcus oriscaviae sp. nov., to describe this bacterium.

## RESULTS

### Patient.

A 24-year-old Chinese man, with good past health, was admitted because of a guinea pig bite, resulting in a 2 mm bite mark at the dorsum of the left hand between the heads of the second and third metacarpals. There was a hematoma and purulent discharge a few hours after bitten by a guinea pig. Two guinea pigs (GP1 and GP2, Fig. S1 in the supplemental material) were purchased from a local pet store as pets a few years ago. They were fed with grass or vegetables purchased from the pet store and were apparently healthy all along. In the evening on the day of admission, the patient was bitten by one of the two guinea pigs (GP1) while he was trying to stop the two guinea pigs from fighting. The swelling increased in the next few hours. There was no numbness or any symptoms suggestive of compartment syndrome. Examination showed that the patient was afebrile and vital signs were normal. Neurological examination did not reveal any sensory or motor deficit. The complete blood count and liver and renal function tests were within normal limits. Radiographic examination did not reveal any fracture. The pus from the wound was sent for bacterial culture. Intravenous amoxicillin-clavulanate 1.2 g q8h was commenced. After 24 h of incubation, a Gram-positive aerobic nonsporulating coccus (the strain tentatively named HKU75^T^) was isolated from the purulent discharge. The swelling gradually subsided and the patient was discharged on day 3. The patient was continued with oral amoxicillin-clavulanate 1 g q12 h for four more days. The patient as well as the two guinea pigs remained asymptomatic at the time of writing, 10 months after discharge.

### Phenotypic characterizations.

HKU75^T^ grew on sheep blood agar as alpha-hemolytic, and gray colonies of 0.5–1 mm in diameter after 24 h of incubation at 37°C in aerobic environment (Fig. S2). Growth enhancement was observed under 5% CO_2_ conditions. The strain did not grow on bile esculin agar, or in 6.5% NaCl. Serogrouping results showed that HKU75^T^ reacted with antisera of both Lancefield groups D and G. It was resistant to optochin, but was sensitive to bacitracin and was non-motile at both 25°C and 37°C. MALDI-TOF MS identified HKU75^T^ as S. suis, with a score of 1.86. The biochemical profile of strain HKU75^T^ is shown in Table 1; it was Voges-Proskauer test negative. It produced leucine, alanine, and tyrosine arylamidase, but did not produce catalase or urease as determined by the Vitek 2 system. It hydrolyzed esculin (API 20) and arginine (Vitek 2 and API 20), and utilized lactose (Vitek 2 and API 20), mannitol (Vitek 2 and API 20), salicin (Vitek 2), sucrose (Vitek 2), trehalose (Vitek 2 and API 20), inulin (API20), mannose (Vitek 2), maltose (Vitek 2), starch (API20), glycogen (API20), amygdalin (Vitek 2), and galactose (Vitek 2). The Vitek 2 system identified HKU75^T^ as “unidentified organism.” The API system showed that its identity was most likely S. suis I with 85.4% confidence. It was sensitive to penicillin (MIC ≤0.016 μg/mL), ceftriaxone, cefepime, levofloxacin, clindamycin, erythromycin, ofloxacin, tetracycline, and vancomycin.

**TABLE 1:** Biochemical profile of *S. oriscaviae* HKU75^T^ and S. suis S735^T^ by Vitek 2 and API 20 STREP^*a*^

Biochemical reaction or enzyme	Result by testing method			
	Vitek 2		API 20 STREP	
	S. suis	*S. oriscaviae*	S. suis	*S. oriscaviae*
Resistance to bacitracin	−	−		
Resistance to optochin	+	+		
Growth in 6.5% NaCl	−	−		
Esculin hydrolysis			+	+
Hippurate hydrolysis			−	−
Arginine hydrolysis	+	+	+	+
Urease	−	−		
Voges-Proskauer test			−	−
Resistance to novobiocin	−	−		
Resistance to polymyxin B	+	+		
Utilization of:				
Lactose	+	+	+	+
Mannitol	−	+	−	+
Raffinose	+	−	+	−
Salicin	+	+		
Sorbitol	+ (v)	−	−	−
Sucrose	+	+		
Trehalose	+	+	+	+
Arabinose			−	−
Pullulan	−	−		
Inulin			+	+
Ribose	−	−	−	−
Xylose	−	−		
D-mannose	+	+		
Maltose	+	+		
Starch			+	+
Glycogen			+	+
Methyl-β-D-glucopyranoside	+	−		
Cyclodextrin	−	−		
D-amygdalin	−	+		
D-galactose	+	+		
N-acetyl-D-glucosamine	+	−		
Pyrrolidonylarylamidase	−	−	−	−
α-galactosidase	+	−	+	−
β-glucuronidase	− (v)	−	+	−
β-galactosidase	−	−	−	−
Leucine arylamidase	+	+		
Leucine aminopeptidase			+	+
Alkaline phosphatase			−	−
Alanine-phenylalanine-proline arylamidase	+	−		
Phosphatidylinositol phospholipase C	−	−		
α-glucosidase	+	−		
L-aspartate arylamidase	−	−		
β-galactopyranosidase	−	−		
α-mannosidase	−	−		
Phosphatase	−	−		
L-proline arylamidase	−	−		
β-glucuronidase	+	−		
Alanine arylamidase	+	+		
Tyrosine arylamidase	+	+		
L-lactate alkalinization	−	−		
O/129 resistance (comp. vibrio)	−	−		
Arginine dihydrolase 2	+	−		

a −, negative; +, positive; (v), variable from previously reported strains (51). All data were obtained by the same methodology using the same culture conditions.

### Molecular characterizations.

**FIG 1:**
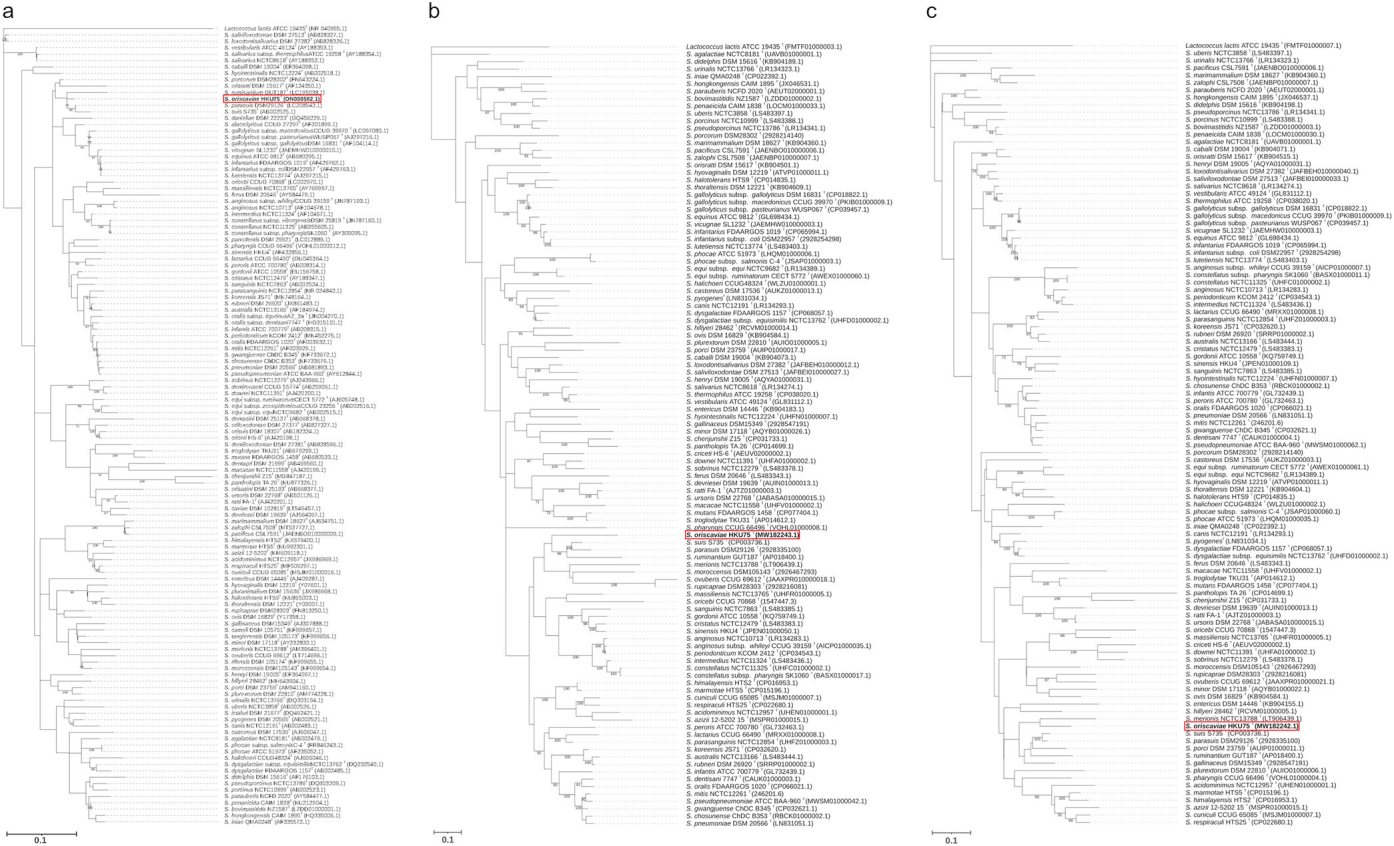
Phylogenetic trees showing the relationship of *S. oriscaviae* HKU75^T^ to its closely related Streptococcus species. The tree was inferred from the sequence data of (a) the 16S rRNA gene, (b) the partial *groEL* gene, and (c) the partial *rpoB* gene by the maximum-likelihood method using Kimura’s two parameter correction (16S rRNA) and general time reversible (*groEL* and *rpoB*) models, with Lactococcus lactis ATCC 19435^T^ as the outgroup. The scale bar indicates the estimated number of substitutions per base. Numbers at nodes indicate levels of bootstrap support calculated from 1,000 pseudoreplicates (values lower than 70 are not shown). Names and nucleotide accession numbers are given as cited in GenBank/JGI/PATRIC. The accession numbers for L. lactis ATCC 19435^T^ are NR_040955.1 (16S rRNA), FMTF01000003 (*groEL*), and FMTF01000007 (*rpoB*).

PCR (PCR) of the 16S rRNA, partial *groEL*, and partial *rpoB* genes of HKU75^T^ yielded DNA products with lengths of approximately 1,500, 600, and 700 bp, respectively. Pairwise alignment showed that the complete 16S rRNA sequence of HKU75^T^ possessed a 98.3% nucleotide identity to Streptococcus parasuis SUT-286^T^, a 97.8% nucleotide identity to Streptococcus porcorum DSM 28302^T^, a 96.7% nucleotide identity to Streptococcus gordonii ATCC 10558^T^, and 96.3% nucleotide identity to S. suis S735^T^; the partial *groEL* sequence of HKU75^T^ possessed an 81.8% nucleotide identity to S. suis S735^T^, an 81.7% nucleotide identity to *S. parasuis* SUT-286^T^, an 81.4% nucleotide identity to Streptococcus dentisani 7747^T^, and a 78.2% nucleotide identity to Streptococcus rubneri DSM 26920^T^; the partial *rpoB* sequence of HKU75^T^ possessed an 89.8% nucleotide identity to S. suis S735^T^, an 89.5% nucleotide identity to *S. parasuis* SUT-286^T^, an 88.8% nucleotide identity to Streptococcus gallinaceus CIP 107087^T^, and an 87.6% nucleotide identity to Streptococcus merionis NCTC 13778^T^ (Fig. 1). These results suggested that phylogenetic analyses using sequences of single gene loci, 16S rRNA, *groEL*, and *rpoB*, failed to determine the taxonomic position of HKU75^T^ within the genus Streptococcus.

### Screening of HKU75^T^ in guinea pigs.

**FIG 2:**
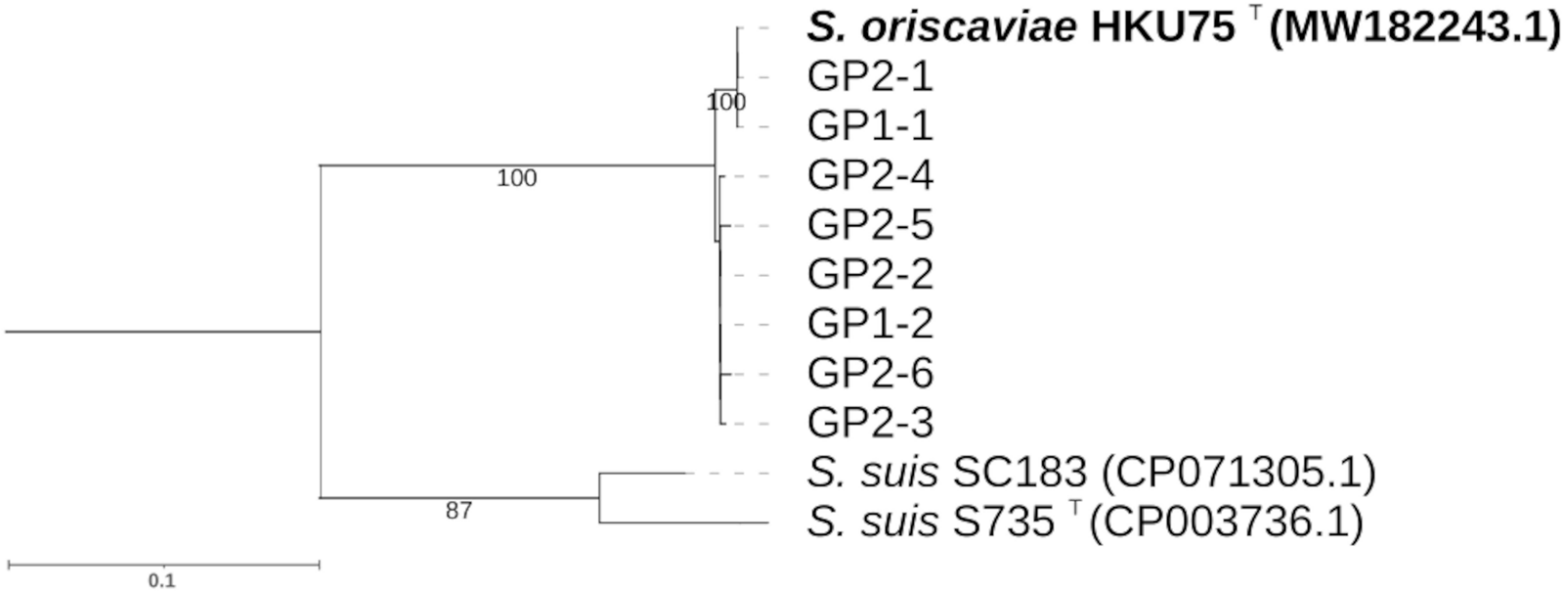
Phylogenetic analysis of *groEL* sequences detected in the oral swabs of guinea pigs GP-1 and GP-2. The tree was constructed by the maximum-likelihood method using the Tamura 3-parameter model with L. lactis ATCC 19435^T^ as the outgroup. A total of 588 nucleotide positions was included in the analysis. Bootstrap values were calculated as percentages from 1,000 pseudoreplicates (values lower than 70 are not shown). The scale bar indicates the estimated number of substitutions per 100 bases. Names and GenBank nucleotide accession numbers are given as cited in GenBank. The accession number for the *groEL* gene sequence of L. lactis ATCC 19435^T^ is FMTF01000003.

Direct cultures of the two oral swabs of GP1 and GP2 failed to isolate HKU75^T^. PCR targeting the partial *groEL* gene fragment of HKU75^T^ yielded DNA products with lengths of approximately 600 bp in DNA samples extracted from the two oral swabs of the guinea pigs. Sequencing and phylogenetic analysis of the clones from each sample showed that GP1 contained two sequence types (GP1-1 and GP1-2) while GP2 contained six sequence types (GP2-1, GP2-2, GP2-3, GP2-4, GP2-5, and GP2-6). There were five (0.85%) nucleotide differences between GP1-1 and GP1-2, and five to seven (0.85–1.19%) nucleotide differences among the six sequence types of GP2. GP1-1 was identical to GP2-1, and both types showed 100% nucleotide identity to the *groEL* gene sequence of HKU75^T^; the next closest match was S. suis SC183, which only shared 85.20% of the nucleotide identity (Fig. 2).

### Comparative genomic characterizations.

**FIG 3:**
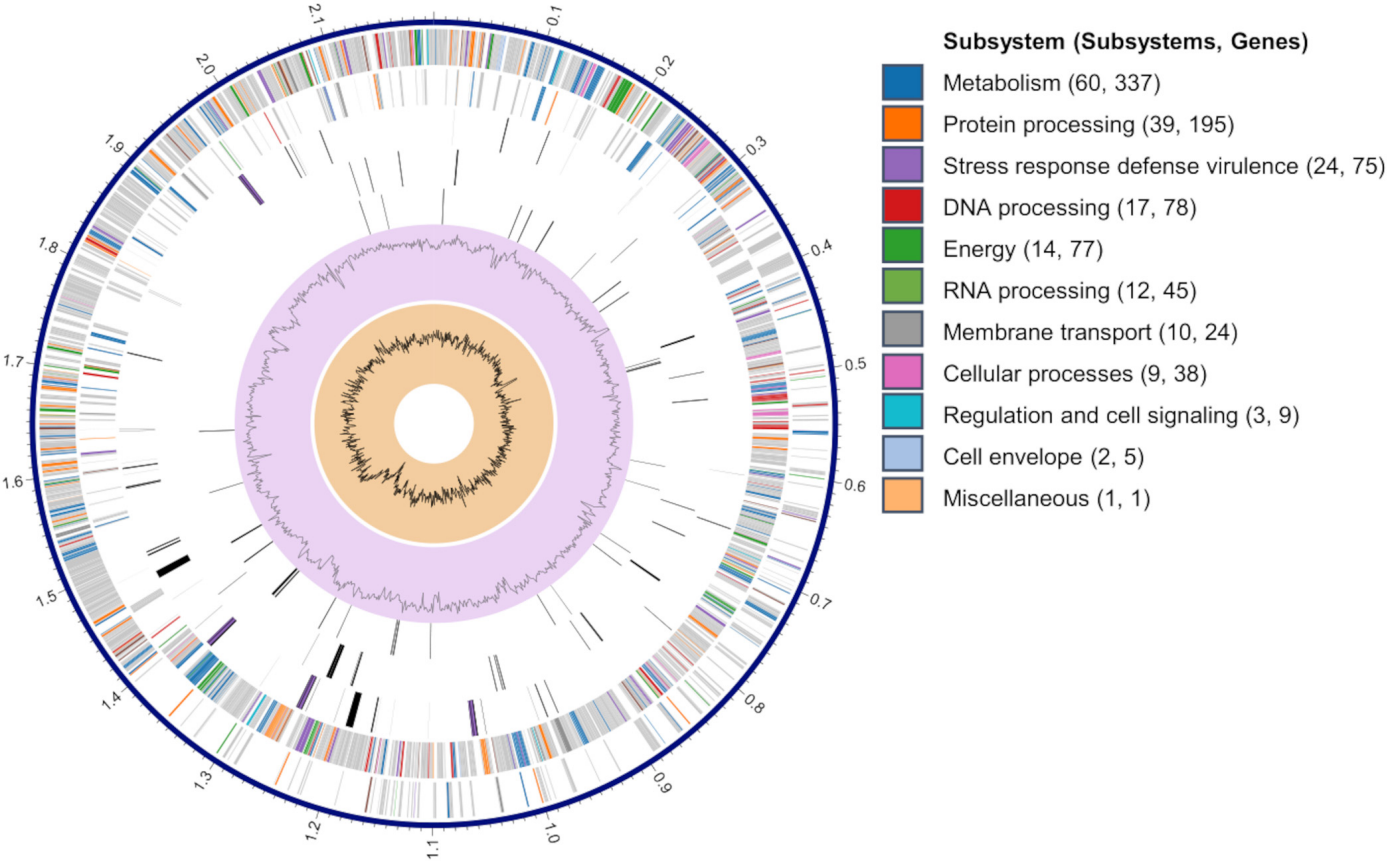
A circular genomic map of *S. oriscaviae* HKU75^T^, showing a circular graphical display of the distribution of *S. oriscaviae* genome annotations. From outer to inner rings: contigs, CDS on the forward strand, CDS on the reverse strand, RNA genes, CDS with homology to known antimicrobial resistance genes, CDS with homology to know virulence factors, G+C content, and GC skew. The colors of the CDSs on the forward and reverse strands indicate the subsystems that these genes belong to.

The *de novo* assembly conducted using both Illumina and Nanopore reads generated one contig, giving a total genome size of 2,197,335 bp (N50 = 2,197,335 bp, 348× coverage) with an average G+C content of 44.1% (Table 2). The contig was submitted to NCBI Prokaryotic Genome Annotation Pipeline (PGAP) for annotation, resulting in 2,119 protein-coding sequences (CDSs), 4 rRNA operons, and 57 tRNA-encoding genes (Table 2). The result of subsystem analysis is summarized in Fig. 3 and Table 3. An *in-silico* genome-to-genome comparison showed that HKU75^T^ was closest to Streptococcus porcorum DSM 28302^T^ (average nucleotide identity [ANI] value of 84.7%), followed by Streptococcus ferus DSM 20646^T^ (ANI value of 84.2%) and Streptococcus porci DSM 23759^T^ (ANI value of 83.9%), with ANI values <95%, a threshold value for species boundary (12, 13) (Table 2; Table S1). This supports that HKU75^T^ should be proposed as a novel Streptococcus species, tentatively named Streptococcus oriscaviae HKU75^T^. Further comparative genomic analyses with closest Streptococcus species revealed that HKU75^T^ were typical of members of Streptococcus (Table 2).

**TABLE 2:** Comparative genomic analysis between *S. oriscaviae* HKU75^T^ and the next 19 ANI closest Streptococcus genomes

Species	Genome size (bp)	G+C content (%)	No. of proteins	No. of rRNA (5S, 16S, 23S)	No. of tRNA	ANI (%)	G+C content difference (%)	GenBank accession no.
Streptococcus *oriscaviae* HKU75^T^	2,197,335	44.1	2,119^*a*^	12^*a*^	57^*a*^			GCA_018137985.1
Streptococcus porcorum DSM 28302^T^	1,899,330	38.2	1,846	3	41	84.7	5.8	2928214140 ^*b*^
Streptococcus ferus DSM 20646^T^	1,872,314	42.8	1,819	15	64	84.2	1.2	GCA_900475025.1
Streptococcus porci DSM 23759^T^	2,289,031	40.8	2,297	6	32	83.9	3.3	GCA_000423765.1
Streptococcus orisratti DSM 15617^T^	2,415,121	38.5	2,350	3	26	83.9	5.5	GCA_000380105.1
Streptococcus pseudoporcinus NCTC13786^T^	2,156,061	37.1	2,004	18	67	83.6	6.9	GCA_900637075.1
Streptococcus dysgalactiae subsp. *equisimilis* NCTC13762^T^	2,285,205	39.5	2,324	18	69	83.5	4.5	GCA_900459095.1
Streptococcus agalactiae NCTC8181^T^	2,448,053	35.7	2,555	21	80	83.3	8.3	GCA_900458965.1
Streptococcus plurextorum DSM 22810^T^	2,100,658	41.1	2,091	6	31	83.3	3.0	GCA_000423745.1
Streptococcus parauberis NCFD 2020^T^	2,164,480	35.5	2,129	15	58	82.9	8.6	GCA_000187935.2
Streptococcus urinalis NCTC13766^T^	2,144,000	34.3	2,212	18	70	82.8	9.8	GCA_900636885.1
Streptococcus canis NCTC12191^T^	2,084,744	39.9	1,974	18	67	82.7	4.2	GCA_900636575.1
Streptococcus uberis NCTC3858^T^	1,975,601	36.6	1,919	18	69	82.6	7.5	GCA_900475595.1
Streptococcus gallolyticus subsp. *pasteurianus* WUSP067^T^	2,149,841	37.3	2,054	18	70	82.4	6.7	GCA_004843545.1
Streptococcus equi subsp. *equi* NCTC9682^T^	2,253,416	41.3	2,303	18	65	82.1	2.8	GCA_900637675.1
Streptococcus pyogenes DSM 20565^T^	1,914,862	38.5	1,899	18	67	82.0	5.6	GCA_002055535.1
Streptococcus suis S735^T^	1,980,887	41.4	1,858	12	56	81.9	2.7	GCA_000294495.1
Streptococcus iniae QMA0248^T^	2,116,570	36.8	2,006	15	58	81.9	7.2	GCA_002220115.1
Streptococcus dysgalactiae FDAARGOS 1157^T^	2,151,179	39.3	2,142	18	67	81.8	4.7	GCA_016724885.1
Streptococcus salivarius NCTC8618^T^	2,206,150	40.1	2,001	18	68	81.7	3.9	GCA_900636435.1

a The genome was annotated by PGAP (47).

b The genome was downloaded from JGI (52).

**TABLE 3:** Distributions of predicted coding sequence function and potential virulence genes in the annotated genome of *S. oriscaviae* HKU75^T^

Genome features		
Subsystem^*a*^	No. of subsystems	No. of genes
Class		
Metabolism	60	337
Protein processing	39	195
Stress response, defense, virulence	24	75
DNA processing	17	78
Energy	14	77
RNA processing	12	45
Membrane transport	10	24
Cellular processes	9	38
Regulation and cell signaling	3	9
Cell envelope	2	5
Miscellaneous	1	1
		
Virulence factors^*b*^	Gene	Locus tag
Class		
Adherence	Alpha-glucosyltransferase (*gftA*)^*c*^	INT76_01865
	Collagen binding protein (*cpbA*)	INT76_08030
	Fibronectin-binding proteins (*pavA*)	INT76_02425
	Laminin-binding protein (*lmb*)	INT76_05165
	Sortase A (*srtA*)	INT76_02095
	Streptococcal lipoprotein rotamase A (*slrA*)	INT76_10040
	Streptococcal plasmin receptor/GAPDH (*plr*/*gapA*)	INT76_05895
Enzyme	Hyaluronidase (*hysA*)	INT76_10670
	Streptococcal enolase (*eno*)	INT76_02460
Protease	C5a peptidase (*scpA*/*scpB*)	INT76_05155
	Serine protease (*htrA*/*degP*)	INT76_07235
	Trigger factor (*tig*/*ropA*)	INT76_05170
	Zinc metalloproteinase (*zmpC*)	INT76_08495

a The subsystems was annotated by PATRIC 3.6.9 (53).

b Virulence factors were annotated by VFDB (48).

c Virulence factor was annotated by PATRIC 3.6.9 (53).

**FIG 4:**
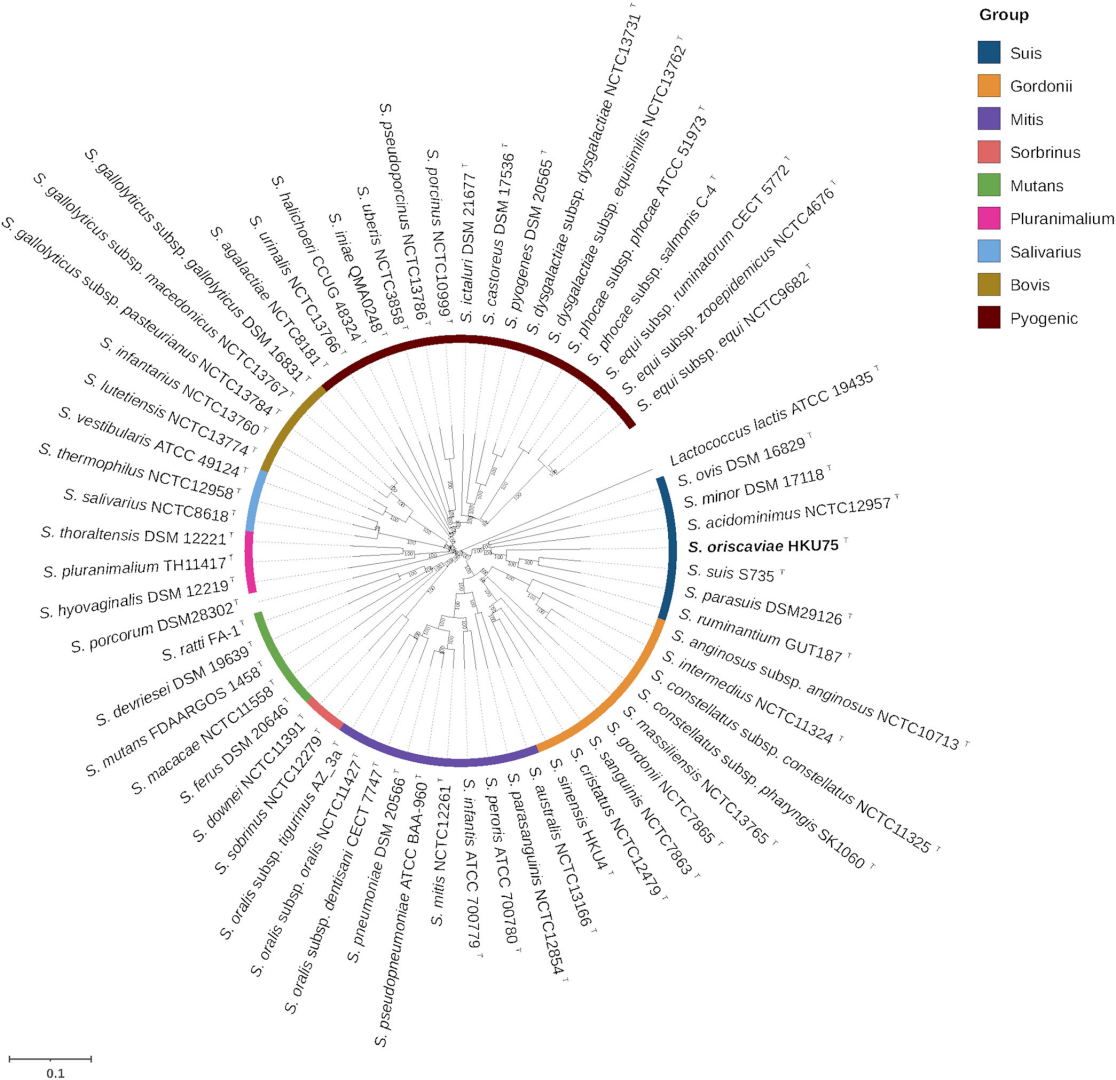
Phylogenetic tree constructed by using concatenated nucleotide sequences of 92 core genes of *S. oriscaviae* HKU75^T^ and its closely related Streptococcus species. The tree was constructed by the neighbor-joining method using MEGA and Lactococcus lactis as the root. The gene names and accession numbers are given as cited in the Database of Clusters of Orthologous Genes (COGs) listed in supplemental Table S3. The Streptococcus genomes were classified into 9 taxonomic groups, namely, Bovis, Gordonii, Mitis, Mutans, Pluranimalium, Pyogenic, Salivarius, Sobrinus, and Suis. The color represents different taxonomic groups. The scale bar corresponds to the mean number of nucleotide substitutions per site on the respective branch. Bootstrap values were calculated as percentages from 1,000 pseudoreplicates (values lower than 70 are not shown).

To elucidate the phylogenetic position of *S*. *oriscaviae* HKU75^T^ among the genus Streptococcus, a multigene-based phylogenomic analysis was performed. The tree based on the concatenated nucleotide sequences of 92 bacterial core genes showed that *S*. *oriscaviae* clustered with members of the Suis group, but not *S. porcorum* (undefined taxonomic group) (Fig. 4). The Suis group currently includes some medically important pathogens such as *S. acidominimus* and *S. ovis*, forming a distinct and well supported phylogenetic clade (Fig. 4). The tree was also able to recover members of the remaining 8 taxonomic groups, including Bovis, Gordonii, Mitis, Mutans, Pluranimalium, Pyogenic, Salivarius, and Sobrinus (Fig. 4), as described in previous studies (14–18).

### Chemotaxonomic characteristics of *S. oriscaviae* HKU75^T^.

Peptidoglycan type of HKU75^T^ was A3α l-lysine-l-alanine, with rhamnose, ribose, and glucose as major cell-wall constituents. Menaquinones and ubiquinones were not detected. The major cellular fatty acids are C16:0 (35.98%) and summed feature 5 (comprising C18:2 ω6,9c/C18:0 ante) (11.38%).

### Potential virulence factors of *S. oriscaviae* HKU75^T^.

Streptococci is commonly found in the oral cavities of pet animals, representing one of the most common genera isolated from animal bite wounds (1). Similar to other streptococci, *S. oriscaviae* HKU75^T^ may possess virulence factors that enable it to colonize the oral cavity of guinea pigs and to establish bite wound infection. The complete genome of *S. oriscaviae* HKU75^T^ identified in this study contained homologs of several virulence genes found in streptococci (Table 3). Examples of these genes include glucosyltransferases (*gtfA*), fibronectin-binding protein (*pavA*), collagen-binding protein (*cpbA*), laminin-binding protein (*lmb*) and enolase (*eno*); these genes are known to be involved in adhesion, colonization, internalization, or invasion (19–24).

## TAXONOMY

### Description of Streptococcus oriscaviae sp. nov.

Streptococcus *oriscaviae* (o.ris.ca'vi.ae. L. neut. n. *os* (gen. *oris*), mouth; N.L. fem. n. *cavia*, a guinea pig (genus *Cavia*); N.L. gen. n. *oriscaviae*, of the mouth of a cavia).

Aerobic. Gram-stain positive. Non-motile. Non-spore-forming. Negative for catalase and urease. Grows best on Columbia agar with 5% defibrinated sheep blood agar. Grows as alpha-hemolytic and gray colonies of 0.5–1 mm in diameter after a 24 h of incubation at 37°C in an aerobic environment. Growth occurs at 37°C but not at 10°C or 42°C. Capable of growing on brain heart infusion agar, nutrient agar, Trypticase soy agar, and chocolate agar. Using the API 20 STREP system, it can assimilate d-mannitol, esculin, d-lactose, d-trehalose, inulin, starch, l-leucine-ß-naphthylamide, l-arginine, and glycogen, but not l-arabinose, d-ribose, d-sorbitol, d-raffinose, pyroglutamic acid-ß-naphthylamide, 6-bromo-2-naphthyl-αD-galactopyranoside, naphthol ASBI-glucuronic acid, 2-naphthyl-ßD-galactopyranoside, or 2-naphthyl phosphate. The peptidoglycan type of HKU75^T^ is A3α l-lysine-l-alanine. Menaquinones and ubiquinones are absent.

The type strain, HKU75^T^ (= CCUG 75141^T^ = JCM 34455^T^), was isolated from the guinea pig bite wound of a patient in Hong Kong. The G+C content of the DNA of the type strain HKU75^T^ was 44.1%. The GenBank accession numbers of the whole genome, 16S rRNA, *groEL*, and *rpoB* genes for the strain HKU75^T^ are CP073084, ON000582, MW182243, and MW182242, respectively.

## DISCUSSION

In this study, we report the isolation of HKU75^T^ in pure culture from the pus collected from a guinea pig bite wound in a healthy young patient. HKU75^T^ is an alpha-hemolytic streptococcus that agglutinates with Lancefield group D and group G antisera. When we first tried to identify HKU75^T^ to the species level, MALDI-TOF MS, the platform we currently used for rapid identification of bacterial isolates in our clinical microbiology laboratory (25–27), showed that the bacterium was most compatible with S. suis, but with a top match score of only 1.86. Therefore, more phenotypic tests were performed using two commercially available kits. One of the kits (API 20 STREP) showed that the most likely identity for HKU75^T^ was S. suis I with 85.4% confidence while the other (Vitek 2) showed that HKU75^T^ was unidentifiable (Table 1). In view of the ambiguous phenotypic profile and inconclusive MALDI-TOF MS results, 16S rRNA and partial *groEL* and *rpoB* gene sequencing were performed. Although the complete 16S rRNA gene of HKU75^T^ was most closely related to that of *S. parasuis* (98.3% sequence identity), partial *groEL* and *rpoB* gene sequence comparison showed that it was more closely related to S. suis (81.8% and 89.8% sequence identity respectively) than *S. parasuis* (81.7% and 89.5% sequence identity respectively) (Fig. 2). Accurate identification of the bacterium was not only of biological interest but also important clinically because if it is a strain of S. suis, the patient could have a significant risk of meningitis and hearing loss (28–31). Finally, whole genome sequencing was performed and intergenomic distance determined by ANI revealed that there was less than 85% identity between the genome of HKU75^T^ and those of all other known species in the Streptococcus genus, confirming that HKU75^T^ is a novel Streptococcus species, which we propose to be named *S. oriscaviae*.

The oral cavity of guinea pigs is presumably a reservoir of *S. oriscaviae*. Although guinea pigs are common household pets of children, unlike dog bite and cat bite wound infections, guinea pig bite wound infections were uncommonly reported. In the literature, guinea pig bite wound infections caused by Haemophilus influenzae and *Pasteurella* species have been described (32, 33). In the present study, when *S. oriscaviae* was first isolated from the pus collected from the patient’s wound, we suspected that the bacterium could have originated from the oral cavities of the guinea pigs owned by the patient, as the wound was inflicted by a bite by the pet. Since *S. oriscaviae* was highly susceptible to most antibiotics, no selective medium could be successfully generated (data not shown). Therefore, we tried to directly amplify the *groEL* gene of the bacterium from DNA extracts obtained from the oral cavities of the two guinea pigs at the patient’s home using specific PCR primers (Fig. S1). Results showed that *groEL* gene sequences (GP1-1 and GP2-1) identical to that of the HKU75^T^ isolate could be amplified from the oral cavities of both guinea pigs (Fig. 2), indicating that guinea pigs are likely a reservoir of *S. oriscaviae*. It is also notable that *groEL* gene sequences (GP1-2 and GP2-2 to GP2-6) that differed by 5–7 bases from HKU75^T^ were also present (Fig. 2), suggesting that multiple strains of *S. oriscaviae* or other very closely related streptococci may be present in the oral cavities of the guinea pigs.

*S. oriscaviae* is a member of the Suis group in the Streptococcus genus. With the advancement of sequencing technologies and accumulation of more and more complete bacterial genomes, streptococci can now be classified using the core gene sequences of their genomes (14–18). In general, the conclusions drawn from phenotypic classification match quite well with those obtained from genomic classification. For example, the beta-hemolytic streptococci are clustered and form a Pyogenic group; and members of the unique *S. milleri* group, namely, *S. anginosus*, S. intermedius and *S. constellatus*, are also closely related to each other phylogenetically. Previously, we have also used its genome sequence to characterize *S. sinensis*, which we discovered and found to be a cause of infective endocarditis (34), and observed that what we suspected about its phylogenetic position from its phenotypic characteristics could be confirmed by genome classification (35–37). As for *S. oriscaviae* in the present study, biochemically the best match was S. suis. When MALDI-TOF MS was used, it was also most closely related to S. suis. 16S rRNA and *groEL*/*rpoB* gene sequence analysis showed that it was most closely related to *S. parasuis* and S. suis respectively (Fig. 1). Genome classification using concatenated sequences of 92 bacterial core genes also showed that it was most closely related to the S. suis/*S. parasuis*/*S. ruminatium* cluster (Fig. 4), confirming that it is a member of the Suis group. It is of note that both *S. parasuis* and *S. ruminatium* were previously different serotypes of S. suis but were recently reclassified using data from molecular and genetic tests. *S. parasuis* was formerly S. suis serotype 20, 22, and 26 but was reclassified as *S. parasuis* in 2015, whereas *S. ruminatium* was formerly S. suis serotype 33 but was reclassified as *S. ruminatium* in 2017 (38, 39). We speculate that some of the reported S. suis isolated from clinical specimens may in fact be *S. parasuis*, *S. ruminatium*, or *S. oriscaviae*. Further studies on these isolates will reveal the relative clinical importance of these Streptococcus species.

## MATERIALS AND METHODS

### Patient and strains.

Clinical specimens were collected and handled according to standard protocols as described previously (40), and were cultured on sheep blood agar at 37°C with 5% CO_2_ to obtain the case isolate HKU75^T^. Clinical data were collected by retrieving and analyzing the hospital record of the patient. The type strain of S. suis S735^T^ was originally isolated from cases of bacteremia/meningitis in piglets; and it was obtained from the Biological Resource Center of Institut Pasteur, France. The quality control strain, Streptococcus pneumoniae ATCC 49619, was obtained for the susceptibility test from the American Type Culture Collection, USA. The study was approved by the Institute Review Board of the University of Hong Kong/Hospital Authority Hong Kong West Cluster (reference number: UW 16–365).

### Microbiological methods and phenotypic characterizations.

Bacterial cultures and phenotypic identification were performed according to standard protocols (40). In addition, the Vitek 2 System (bioMérieux, Marcy l’Etoile, France) and the API system (20 STREP) (bioMérieux, France) were used to identify the bacterial isolate in this study. Lancefield serogrouping was performed using Streptex (Murex Biotech Ltd., Dartford, United Kingdom) according to the manufacturer’s instructions. MALDI-TOF MS was performed by the direct transfer method, as described previously, with modifications (41). This was conducted using the Microflex LT system with MALDI Biotyper 3.0 and Reference Library V.3.1.2.0 (Bruker Daltonik). Antibiotic susceptibility testing was performed using the Etest method for penicillin and the Kirby Bauer disk diffusion method for the other antibiotics; the results were interpreted according to the Clinical and Laboratory Standards Institute for alpha-hemolytic streptococci.

### 16S rRNA, partial groEL, and partial rpoB gene sequencing; sequence identity analyses and phylogenetic analyses.

Extractions of bacterial DNA, PCR, and sequencing of the 16S rRNA, *groEL*, and *rpoB* genes for the case isolate HKU75^T^ were carried out following the methods outlined in a previous publication, with slight modifications (42, 43). The primer pairs LPW40131 (5′-CTAAGGCCCCACAAGACCTC-3′) and LPW40132 (5′-CAGAGTGCTTAGCCGGACAA-3′), LPW15046 (5′-GAHGTNGTiGAAGGiATGCA-3′) and LPW15047 (5′-ATTTGRCGiAYWGGYTCTTC-3′), and LPW38616 (5′-TCGTCAACCATGTGGTGA-3′) and LPW38617 (5′-GGGCCTGAAGAAATCACC-3′) were used for the 16S rRNA, *groEL* and *rpoB* genes, respectively, for the respective PCR and DNA sequencing. The DNA sequences obtained, together with those of other closely related species accessioned in the DDBJ/ENA/GenBank/JGI/PATRIC databases (Table S2), were then compared by pairwise alignment, using MEGA 11 (version 11.0.11) (44). These sequences were also analyzed via multiple sequence alignment, using ClustalW (45). Tests for substitution models and the phylogenetic tree construction were performed using the maximum likelihood method, using MEGA 11 (version 11.0.11) (44). Phylogenetic analyses included 1,287, 666, and 628 nucleotide positions of the 16S rRNA, partial *groEL*, and partial *rpoB* sequences, respectively.

### Sample collection from guinea pigs and identification of HKU75^T^.

Two oral swabs were prospectively collected from each of the two guinea pigs (GP1 and GP2) (Fig. S1). Before sample collection, the guinea pigs were not allowed to eat or drink for 30 min. Briefly, the swabs (Oxoid) were inserted into the mouths of the guinea pigs and slowly twirled, and the swab was then rubbed across the tooth surface/mucosa to collect the samples. The swabs were immediately stored in Amies agar gel and transported to a laboratory at ambient temperature. One swab was cultured on sheep blood agar at 37°C with 5% CO_2_ for 48 h. The genus identities of all suspected Streptococcus-like isolates were confirmed by MALDI-TOF MS, as described above. Another oral swab was subjected to direct DNA extraction using the alkaline lysis method, as described previously (42). The HKU75^T^ in the DNA extracts, as well as in all Streptococcus species isolated from the swab samples, was detected using PCR targeting its partial 588-bp fragment of the *groEL* gene. The obtained sequences were compared with the *groEL* gene sequence of HKU75^T^.

As double or multiple nucleotide peaks were observed in the sequencing results, the corresponding purified PCR product was cloned into the pCR-II-TOPO vector (Invitrogen), according to manufacturer’s instructions. Eight and 11 clones were selected for GP-1 and GP-2 respectively. Both strands of each clone were sequenced using the primers 5′-GTAAAACGACGGCCAG-3′ and 5′-CAGGAAACAGCTATGAC-3′. The sequences of the clones were compared with the HKU75^T^ genome and other *groEL* gene sequences of Streptococcus species available in the GenBank database. Phylogenetic analysis was performed as described above, using the 588 nucleotide positions of the partial *groEL* sequences.

### Chemotaxonomic characterizations.

Analysis of peptidoglycan, cell-wall sugars, quinones, and fatty acid were carried out by DSMZ Services, Leibniz-Institut DSMZ-Deutsche Sammlung von Mikroongranismen und Zellkulturen GmbH, Braunschweig, Germany.

### Whole genome sequencing and hybrid genome assembly.

The complete genome of the case isolate HKU75^T^ was sequenced using Illumina and Oxford Nanopore technologies. Genomic DNA was extracted from an overnight culture (37°C) grown on blood agar using a genomic DNA purification kit (Qiagen, Hilden, Germany), as described previously (42). The Illumina DNA library was prepared using a Nextera XT DNA Sample Prep Kit (Illumina, San Diego, CA, USA) and was sequenced on a NovaSeq 6000 instrument (run type: PE151 bp). The ONT long-read library was prepared with SQK-RAD004 rapid sequencing kit (Oxford Nanopore Technologies, Oxford, UK) according to the manufacturer’s instructions, and sequenced on a MinION sequencer. Illumina reads and Oxford Nanopore MinION reads were assembled by Unicycler/0.4.8 to obtain the whole genome of HKU75^T^.

### Genome sequence analyses.

Intergenomic distances (i.e., ANI values) between the proposed novel species (i.e., the case isolate HKU75^T^) and the type and reference strains of the corresponding closest species were calculated using the web service available at http://enve-omics.ce.gatech.edu/ani/ (46). In addition to the case isolate HKU75^T^, which was sequenced to completion as part of this study, the remaining 104 complete genome sequences of 104 Streptococcus species were downloaded from NCBI, JGI, and PATRIC databases (Table S2). The G+C content of HKU75^T^ was determined based on the genome sequence. Predictions of protein coding regions and automatic functional annotations were performed using the PGAP (47). Virulence genes were identified by using the Virulence Factor Database (VFDB) (48). Comparative genomic analysis between HKU75^T^ and the next 19 ANI closest Streptococcus genomes was performed using the Type Strain Genome Server (49).

### Phylogenomic characterization.

To determine the phylogenetic position of *S*. *oriscaviae* HKU75^T^ among the current 9 taxonomic groups within the genus Streptococcus (14), a multigene-based phylogenomic treeing approach based on concatenated nucleotide sequences of 92 bacterial core genes was used (Table S3). This approach has been shown to be useful for phylogenetic delineation of bacterial species in previous studies (14–17). The alignment of concatenated 92 core genes of 62 Streptococcus genomes and one *Lactococcus* genome was first generated using an up-to-date bacterial core gene (UBCG) pipeline (https://www.ezbiocloud.net/tools/ubcg) with the default parameters as described by Na et al. (Table S4) (50). The Neighbor-joining tree was constructed using MEGA 11 (version 11.0.11) (44).

### Data availability.

The whole genome sequence of HKU75^T^ has been deposited at GenBank under the accession CP073084. The version described in this article is CP073084. The 16S rRNA, *rpoB*, and *groEL* gene sequences of HKU75^T^ have been deposited at GenBank under the accession numbers ON000582, MW182242, and MW182243 respectively.
