# Supplementary material for: Streptococcus oriscaviae sp. nov. Infection Associated with Guinea Pigs
Source: Microbiol Spectr. 2022 May 5;10(3):e00014-22. doi: 10.1128/spectrum.00014-22 (PMC9241640; doi:10.1128/spectrum.00014-22)
Supplement: SUPPLEMENTAL FILE 1 — Supplemental material. Download spectrum.00014-22-s001.pdf, PDF file, 0.4 MB [file spectrum.00014-22-s001.pdf]

Table S1. The pairwise comparison of ANI values between *S. oriscaviae* HKU75^T and 104 *Streptococcus* genomes.

Species	ANI (%)
Streptococcus porcorum DSM28302^T	84.7
Streptococcus ferus DSM 20646^T	84.2
Streptococcus porci DSM 23759^T	83.9
Streptococcus orisratti DSM 15617^T	83.9
Streptococcus pseudoporcinus NCTC13786^T	83.6
Streptococcus dysgalactiae subsp. *equisimilis* NCTC13762^T	83.5
Streptococcus agalactiae NCTC8181^T	83.3
Streptococcus plurextorum DSM 22810^T	83.3
Streptococcus parauberis NCFD 2020^T	82.9
Streptococcus urinalis NCTC13766^T	82.8
Streptococcus canis NCTC12191^T	82.7
Streptococcus uberis NCTC3858^T	82.6
Streptococcus gallolyticus subsp. *pasteurianus* WUSP067^T	82.4
Streptococcus equi subsp. *equi* NCTC9682^T	82.1
Streptococcus pyogenes DSM 20565^T	82.0
Streptococcus suis S735^T	81.9
Streptococcus iniae QMA0248^T	81.9
Streptococcus dysgalactiae FDAARGOS 1157^T	81.8
Streptococcus salivarius NCTC8618^T	81.7
Streptococcus lutetiensis NCTC13774^T	81.5
Streptococcus gallolyticus subsp. *gallolyticus* DSM 16831^T	81.5
Streptococcus didelphis DSM 15616^T	81.4
Streptococcus mitis NCTC 12261^T	81.4
Streptococcus koreensis JS71^T	81.3
Streptococcus salivarius subsp. *thermophilus* ATCC 19258^T	81.1
Streptococcus constellatus subsp. *pharyngis* SK1060^T	81.1
Streptococcus hyointestinalis NCTC12224^T	81.0
Streptococcus hyovaginalis DSM 12219^T	81.0
Streptococcus bovimastitidis NZ1587^T	80.9
Streptococcus infantarius FDAARGOS 1019^T	80.9
Streptococcus intermedius NCTC11324^T	80.9
Streptococcus porcinus NCTC10999^T	80.9
Streptococcus criceti HS-6^T	80.8
Streptococcus halotolerans HTS9^T	80.8
Streptococcus phocae ATCC 51973^T	80.7
Streptococcus parasuis DSM 29126^T	80.7
Streptococcus himalayensis HTS2^T	80.6
Streptococcus equinus ATCC 9812^T	80.6
Streptococcus sobrinus NCTC12279^T	80.6
Streptococcus phocae subsp. *salmonis* C-4^T	80.6
Streptococcus parasanguinis NCTC12854^T	80.6

Streptococcus castoreus DSM 17536^{T}	80.5
Streptococcus minor DSM 17118^{T}	80.5
Streptococcus australis NCTC13166^{T}	80.5
Streptococcus pneumoniae DSM 20566^{T}	80.4
Streptococcus oralis FDAARGOS 1020^{T}	80.4
Streptococcus cristatus NCTC12479^{T}	80.3
Streptococcus troglodytae TKU31^{T}	80.3
Streptococcus equi subsp. *ruminatorum* CECT 5772^{T}	80.2
Streptococcus henryi DSM 19005^{T}	80.2
Streptococcus ruminantium GUT187^{T}	80.2
Streptococcus massiliensis NCTC13765^{T}	80.2
Streptococcus sanguinis NCTC7863^{T}	80.1
Streptococcus hongkongensis CAIM 1895^{T}	80.1
Streptococcus mutans FDAARGOS 1458^{T}	80.1
Streptococcus thoraltensis DSM 12221^{T}	80.0
Streptococcus hillyeri 28462^{T}	80.0
Streptococcus saliviloxodontae DSM 27513^{T}	80.0
Streptococcus constellatus NCTC11325^{T}	79.9
Streptococcus halichoeri CCUG48324^{T}	79.8
Streptococcus gordonii ATCC 10558^{T}	79.8
Streptococcus pacificus CSL7591^{T}	79.8
Streptococcus gwangjuense ChDC B345^{T}	79.8
Streptococcus lactarius CCUG 66490^{T}	79.8
Streptococcus pantholopis TA 26^{T}	79.7
Streptococcus zalophi CSL7508^{T}	79.7
Streptococcus peroris ATCC 700780^{T}	79.7
Streptococcus macacae NCTC11558^{T}	79.7
Streptococcus vicugnae SL1232^{T}	79.7
Streptococcus vestibularis ATCC 49124^{T}	79.6
Streptococcus rubneri DSM 26920^{T}	79.6
Streptococcus penaeicida CAIM 1838^{T}	79.6
Streptococcus pseudopneumoniae ATCC BAA-960^{T}	79.6
Streptococcus respiraculi HTS25^{T}	79.6
Streptococcus chenjunshii Z15^{T}	79.6
Streptococcus infantarius subsp. *coli* DSM22957^{T}	79.5
Streptococcus rupicaprae DSM28303^{T}	79.5
Streptococcus pharyngis CCUG 66496^{T}	79.5
Streptococcus anginosus NCTC10713^{T}	79.5
Streptococcus downei NCTC11391^{T}	79.5
Streptococcus gallolyticus subsp. *macedonicus* CCUG 39970^{T}	79.4
Streptococcus marmotae HTS5^{T}	79.4
Streptococcus loxodontisalivarius DSM 27382^{T}	79.4
Streptococcus merionis NCTC13788^{T}	79.4
Streptococcus marimammalium DSM 18627^{T}	79.3

Streptococcus infantis ATCC 700779^{T}	79.2
Streptococcus ursoris DSM 22768^{T}	79.2
Streptococcus periodonticum KCOM 2412^{T}	79.2
Streptococcus moroccensis DSM105143^{T}	79.1
Streptococcus caballi DSM 19004^{T}	79.1
Streptococcus entericus DSM 14446^{T}	79.0
Streptococcus acidominimus NCTC12957^{T}	79.0
Streptococcus gallinaceus DSM15349^{T}	79.0
Streptococcus ovuberis CCUG 69612^{T}	78.9
Streptococcus oralis subsp. *dentisani* 7747^{T}	78.8
Streptococcus ovis DSM 16829^{T}	78.8
Streptococcus sinensis HKU4^{T}	78.7
Streptococcus ratti FA-1^{T}	78.6
Streptococcus cuniculi CCUG 65085^{T}	78.4
Streptococcus chosunense ChDC B353^{T}	78.4
Streptococcus azizii 12-5202^{T}	78.3
Streptococcus devriesei DSM 19639^{T}	78.1
Streptococcus anginosus subsp. *whileyi* CCUG 39159^{T}	78.0
Streptococcus oricebi CCUG 70868^{T}	77.9

Table S2. Single gene locus and genome sequences used for analyses in the present study.

Species	GenBank accession numbers			
	16S rRNA	*groEL*	*rpoB*	Genome
Streptococcus mitis NCTC 12261^T	AF003929.1	[a]246201	[a]246201	[a]246201.6
Streptococcus oricebi CCUG 70868^T	LC002970.1	[a]1547447	[a]1547447	[a]1547447.3
Streptococcus moroccensis DSM105143^T	KF999654.1	[b]2926467293	[b]2926467293	[b]2926467293
Streptococcus porcorum DSM28302^T	FN643224.1	[b]2928214140	[b]2928214140	[b]2928214140
Streptococcus rupicaprae DSM28303^T	FN813250.1	[b]2928216081	[b]2928216081	[b]2928216081
Streptococcus infantarius subsp. *coli* DSM22957^T	AF429763.1	[b]2928254298	[b]2928254298	[b]2928254298
Streptococcus parasuis DSM 29126^T	LC208542.1	[b]2928335100	[b]2928335100	[b]2928335100
Streptococcus gallinaceus DSM15349^T	AJ307888.1	[b]2928547191	[b]2928547191	[b]2928547191
Streptococcus parauberis NCFD 2020^T	AY584477.1	AEUT02000001.1	AEUT02000001.1	GCA_000187935.2
Streptococcus criceti HS-6^T	AJ420198.1	AEUV02000002.1	AEUV02000002.1	GCA_000187975.3
Streptococcus anginosus subsp. *whileyi* CCUG 39159^T	JN787193.1	AICP01000035.1	AICP01000007.1	GCA_000257765.1
Streptococcus ratti FA-1^T	AJ420201.1	AJTZ01000003.1	AJTZ01000003.1	GCA_000286075.1
Streptococcus troglodytae TKU31^T	AB679299.1	AP014612.1	AP014612.1	GCA_002355215.1
Streptococcus ruminantium GUT187^T	LC195038.1	AP018400.1	AP018400.1	GCA_003609975.1
Streptococcus henryi DSM 19005^T	EF364097.1	AQYA01000031.1	AQYA01000031.1	GCA_000376985.1
Streptococcus minor DSM 17118^T	AY232832.1	AQYB01000026.1	AQYB01000022.1	GCA_000377005.1
Streptococcus hyovaginalis DSM 12219^T	Y07601.1	ATVP01000011.1	ATVP01000011.1	GCA_000420785.1
Streptococcus devriesei DSM 19639^T	AJ564067.1	AUIN01000013.1	AUIN01000013.1	GCA_000423725.1
Streptococcus plurextorum DSM 22810^T	AM774228.1	AUIO01000005.1	AUIO01000006.1	GCA_000423745.1
Streptococcus porci DSM 23759^T	AM941160.1	AUIP01000017.1	AUIP01000011.1	GCA_000423765.1
Streptococcus castoreus DSM 17536^T	AJ606047.1	AUKZ01000013.1	AUKZ01000003.1	GCA_000425025.1
Streptococcus equi subsp. *ruminatorum* CECT 5772^T	AJ605748.1	AWEX01000060.1	AWEX01000061.1	GCA_000706805.1
Streptococcus constellatus subsp. *pharyngis* SK1060^T	AY309095.1	BASX01000017.1	BASX01000011.1	GCA_900459125.1

Streptococcus oralis subsp. *dentisani* 7747^{T}	HG315101.1	CAUK01000003.1	CAUK01000004.1	GCA_000382825.1
Streptococcus suis S735^{T}	AB002525.1	CP003736.1	CP003736.1	GCA_000294495.1
Streptococcus pantholopis TA 26^{T}	KU877326.1	CP014699.1	CP014699.1	GCA_001642085.1
Streptococcus halotolerans HTS9^{T}	KU865003.1	CP014835.1	CP014835.1	GCA_001598035.1
Streptococcus marmotae HTS5^{T}	KU992301.1	CP015196.1	CP015196.1	GCA_001623565.1
Streptococcus himalayensis HTS2^{T}	KX679400.1	CP016953.1	CP016953.1	GCA_001708305.1
Streptococcus gallolyticus subsp. *gallolyticus* DSM 16831^{T}	AF104114.1	CP018822.1	CP018822.1	GCA_002000985.1
Streptococcus iniae QMA0248^{T}	AF335572.1	CP022392.1	CP022392.1	GCA_002220115.1
Streptococcus respiraculi HTS25^{T}	MF509297.1	CP022680.1	CP022680.1	GCA_003595525.1
Streptococcus chenjunshii Z15^{T}	MG847187.1	CP031733.1	CP031733.1	GCA_003086355.2
Streptococcus koreensis JS71^{T}	MK748164.1	CP032620.1	CP032620.1	GCA_003627135.1
Streptococcus gwangjuense ChDC B345^{T}	KF733672.1	CP032621.1	CP032621.1	GCA_003627155.1
Streptococcus periodonticum KCOM 2412^{T}	MK452275.1	CP034543.1	CP034543.1	GCA_003963555.1
Streptococcus salivarius subsp. *thermophilus* ATCC 19258^{T}	AY188354.1	CP038020.1	CP038020.1	GCA_010120595.1
Streptococcus gallolyticus subsp. *pasteurianus* WUSP067^{T}	AJ297216.1	CP039457.1	CP039457.1	GCA_004843545.1
Streptococcus infantarius FDAARGOS 1019^{T}	AF429762.1	CP065994.1	CP065994.1	GCA_016127275.1
Streptococcus oralis FDAARGOS 1020^{T}	AF003932.1	CP066021.1	CP066021.1	GCA_016127555.1
Streptococcus dysgalactiae FDAARGOS 1157^{T}	AB002485.1	CP068057.1	CP068057.1	GCA_016724885.1
Streptococcus oriscaviae HKU75^{T}	ON000582.1	MW182243.1	MW182242.1	GCA_018137985.1
Streptococcus mutans FDAARGOS 1458^{T}	AB680533.1	CP077404.1	CP077404.1	GCA_019048645.1
Lactococcus lactis ATCC 19435^{T}	NR_040955.1	FMTF01000003.1	FMTF01000007.1	GCA_900099625.1
Streptococcus equinus ATCC 9812^{T}	AB680295.1	GL698434.1	GL698434.1	GCA_000187265.1
Streptococcus infantis ATCC 700779^{T}	AB008315.1	GL732439.1	GL732439.1	GCA_000187465.1
Streptococcus peroris ATCC 700780^{T}	AB008314.1	GL732463.1	GL732463.1	GCA_000187585.1
Streptococcus vestibularis ATCC 49124^{T}	AY188353.1	GL831112.1	GL831112.1	GCA_000188295.1
Streptococcus ovuberis CCUG 69612^{T}	LT714696.1	JAAXPR010000018.1	JAAXPR010000021.1	GCA_012396585.1
Streptococcus ursoris DSM 22768^{T}	AB501126.1	JABASA010000015.1	JABASA010000015.1	GCA_012843165.1
Streptococcus vicugnae SL1232^{T}	JAEMHW010000010.1	JAEMHW010000003.	JAEMHW010000003.1	GCA_016461705.1

Streptococcus pacificus CSL7591^T	JAENBO010000009.1	JAENBO010000006.1	JAENBO010000006.1	GCA_016481305.1
Streptococcus zalophi CSL7508^T	MT537727.1	JAENBP010000007.1	JAENBP010000007.1	GCA_016481285.1
Streptococcus loxodontisalivarius DSM 27382^T	AB828326.1	JAFBEH010000012.1	JAFBEH010000040.1	GCA_016908645.1
Streptococcus saliviloxodontae DSM 27513^T	AB828327.1	JAFBEI010000027.1	JAFBEI010000033.1	GCA_016908655.1
Streptococcus sinensis HKU4^T	AF432856.1	JPEN01000050.1	JPEN01000109.1	GCA_000767835.1
Streptococcus hongkongensis CAIM 1895^T	HQ335006.1	JX046531.1	JX046537.1	GCA_000785845.1
Streptococcus phocae subsp. *salmonis* C-4^T	FR846243.1	JSAP01000003.1	JSAP01000060.1	GCA_000772915.1
Streptococcus caballi DSM 19004^T	EF364098.1	KB904073.1	KB904071.1	GCA_000379985.1
Streptococcus entericus DSM 14446^T	AJ409287.1	KB904183.1	KB904155.1	GCA_000380025.1
Streptococcus didelphis DSM 15616^T	AF176103.1	KB904189.1	KB904198.1	GCA_000380005.1
Streptococcus marimammalium DSM 18627^T	AJ634751.1	KB904360.1	KB904360.1	GCA_000380045.1
Streptococcus orisratti DSM 15617^T	AF124350.1	KB904501.1	KB904515.1	GCA_000380105.1
Streptococcus ovis DSM 16829^T	Y17358.1	KB904584.1	KB904584.1	GCA_000380125.1
Streptococcus thoraltensis DSM 12221^T	Y09007.1	KB904609.1	KB904604.1	GCA_000380145.1
Streptococcus gordonii ATCC 10558^T	EU156758.1	KQ759749.1	KQ759749.1	GCA_001469295.1
Streptococcus phocae ATCC 51973^T	AF235052.1	LHQM01000006.1	LHQM01000035.1	GCA_001302265.1
Streptococcus pyogenes DSM 20565^T	AB002521.1	LN831034.1	LN831034.1	GCA_002055535.1
Streptococcus pneumoniae DSM 20566^T	AB681893.1	LN831051.1	LN831051.1	GCA_001457635.1
Streptococcus penaeicida CAIM 1838^T	KU212904.1	LOCM01000033.1	LOCM01000030.1	GCA_002887775.1
Streptococcus salivarius NCTC8618^T	AY188352.1	LR134274.1	LR134274.1	GCA_900636435.1
Streptococcus anginosus NCTC10713^T	AF104678.1	LR134283.1	LR134283.1	GCA_900636475.1
Streptococcus canis NCTC12191^T	AB002483.1	LR134293.1	LR134293.1	GCA_900636575.1
Streptococcus urinalis NCTC13766^T	DQ303194.1	LR134323.1	LR134323.1	GCA_900636885.1
Streptococcus pseudoporcinus NCTC13786^T	DQ303209.1	LR134341.1	LR134341.1	GCA_900637075.1
Streptococcus equi subsp. equi NCTC9682^T	AB002515.1	LR134389.1	LR134389.1	GCA_900637675.1
Streptococcus ferus DSM 20646^T	AY584479.1	LS483343.1	LS483343.1	GCA_900475025.1
Streptococcus sobrinus NCTC12279^T	AJ243966.1	LS483378.1	LS483378.1	GCA_900475395.1

Streptococcus cristatus NCTC12479^{T}	AY188347.1	LS483383.1	LS483383.1	GCA_900475445.1
Streptococcus sanguinis NCTC7863^{T}	AB002524.1	LS483385.1	LS483385.1	GCA_900475505.1
Streptococcus porcinus NCTC10999^{T}	AB002523.1	LS483388.1	LS483388.1	GCA_900475415.1
Streptococcus uberis NCTC3858^{T}	AB002526.1	LS483397.1	LS483397.1	GCA_900475595.1
Streptococcus lutetiensis NCTC13774^{T}	AJ297215.1	LS483403.1	LS483403.1	GCA_900475675.1
Streptococcus intermedius NCTC11324^{T}	AF104671.1	LS483436.1	LS483436.1	GCA_900475975.1
Streptococcus australis NCTC13166^{T}	AF184974.1	LS483444.1	LS483444.1	GCA_900476055.1
Streptococcus merionis NCTC13788^{T}	AM396401.1	LT906439.1	LT906439.1	GCA_900187085.1
Streptococcus bovimastitidis NZ1587^{T}	LZDD01000001.1	LZDD01000002.1	LZDD01000003.1	GCA_001885095.1
Streptococcus lactarius CCUG 66490^{T}	GU045364.1	MRXX01000008.1	MRXX01000008.1	GCA_016642265.1
Streptococcus cuniculi CCUG 65085^{T}	MSJM01000016.1	MSJM01000007.1	MSJM01000007.1	GCA_001921845.1
Streptococcus azizii 12-5202^{T}	KM609118.1	MSPR01000015.1	MSPR01000015.1	GCA_001984715.1
Streptococcus pseudopneumoniae ATCC BAA-960^{T}	AY612844.1	MWSM01000042.1	MWSM01000062.1	GCA_002087075.1
Streptococcus equi subsp. *zooepidemicus* CCUG 23256^{T}	AB002516.1	NA	NA	NA
Streptococcus alactolyticus CCUG 27297^{T}	AF201899.1	NA	NA	NA
Streptococcus ictaluri DSM 21677^{T}	DQ462421.1	NA	NA	NA
Streptococcus orisuis DSM 18307^{T}	AB182324.1	NA	NA	NA
Streptococcus dentirousetti CCUG 55774^{T}	AB259061.1	NA	NA	NA
Streptococcus dentapri DSM 21999^{T}	AB469560.1	NA	NA	NA
Streptococcus danieliae DSM 22233^{T}	GQ456229.1	NA	NA	NA
Streptococcus oralis subsp. *tigurinus* AZ_3a^{T}	JN004270.1	NA	NA	NA
Streptococcus orisasini DSM 25193^{T}	AB668377.1	NA	NA	NA
Streptococcus dentasini DSM 25137^{T}	AB668378.1	NA	NA	NA
Streptococcus constellatus subsp. *viborgensis* DSM 25819^{T}	JN787160.1	NA	NA	NA
Streptococcus pluranimalium DSM 15636^{T}	JX986968.1	NA	NA	NA
Streptococcus oriloxodontae DSM 27377^{T}	AB827327.1	NA	NA	NA
Streptococcus tangierensis DSM 105173^{T}	KF999656.1	NA	NA	NA
Streptococcus cameli DSM 105751^{T}	KF999657.1	NA	NA	NA

Streptococcus rifensis DSM 105174[T]	KF999655.1	NA	NA	NA
Streptococcus panodentis DSM 29921[T]	LC012889.1	NA	NA	NA
Streptococcus dentiloxodontae DSM 27381[T]	AB828596.1	NA	NA	NA
Streptococcus caviae DSM 102819[T]	LT546457.1	NA	NA	NA
Streptococcus gallolyticus subsp. *macedonicus* CCUG 39970[T]	LC097080.1	PKIB01000009.1	PKIB01000009.1	GCA_002860805.1
Streptococcus chosunense ChDC B353[T]	KF733679.1	RBCK01000002.1	RBCK01000002.1	GCA_003626515.1
Streptococcus hillyeri 28462[T]	MH643904.1	RCVM01000014.1	RCVM01000005.1	GCA_003686955.1
Streptococcus rubneri DSM 26920[T]	JX861483.1	SRRP01000002.1	SRRP01000002.1	GCA_004785935.1
Streptococcus agalactiae NCTC8181[T]	AB002479.1	UAVB01000001.1	UAVB01000001.1	GCA_900458965.1
Streptococcus acidominimus NCTC12957[T]	JX986969.1	UHEN01000001.1	UHEN01000001.1	GCA_900459045.1
Streptococcus downei NCTC11391[T]	AJ420200.1	UHFA01000002.1	UHFA01000002.1	GCA_900459175.1
Streptococcus constellatus NCTC11325[T]	AB355605.1	UHFC01000002.1	UHFC01000002.1	GCA_000474135.1
Streptococcus dysgalactiae subsp. *equisimilis* NCTC13762[T]	DQ232540.1	UHFD01000002.1	UHFD01000002.1	GCA_900459095.1
Streptococcus hyointestinalis NCTC12224[T]	AB002518.1	UHFN01000007.1	UHFN01000007.1	GCA_900459405.1
Streptococcus massiliensis NCTC13765[T]	AY769997.1	UHFR01000005.1	UHFR01000005.1	GCA_900459365.1
Streptococcus macacae NCTC11558[T]	AJ420199.1	UHFV01000002.1	UHFV01000002.1	GCA_900459485.1
Streptococcus parasanguinis NCTC12854[T]	NR_024842.1	UHFZ01000003.1	UHFZ01000003.1	GCA_900459355.1
Streptococcus pharyngis CCUG 66496[T]	VOHL01000012.1	VOHL01000008.1	VOHL01000004.1	GCA_007859195.1
Streptococcus halichoeri CCUG48324[T]	AJ606046.1	WLZU01000001.1	WLZU01000002.1	GCA_009870755.1

[a]Genomes downloaded from PATRIC server (1).

[b]Genomes downloaded from JGI (2).

NA, sequences not available in public databases.

Table S3. Core genes used in the UBCG analysis.

Gene	COG ID	Function
alaS	COG0013	Alanine-tRNA ligase
argS	COG0018	Arginine-tRNA ligase
aspS	COG0173	Aspartate-tRNA ligase
cgtA	COG0536	GTPase ObgE/CgtA
coaE	COG0237	Dephospho-CoA kinase
cysS	COG0215	Cysteine-tRNA ligase
dnaA	COG0593	Chromosomal replication initiator protein DnaA
dnaG	COG0358	DNA primase
dnaX	COG2812	DNA polymerase III subunit gamma
engA	COG1160	GTPase Der
ffh	COG0541	Signal recognition particle protein
fmt	COG0223	Methionyl-tRNA formyltransferase
frr	COG0233	Ribosome-recycling factor
ftsY	COG0552	Signal recognition particle receptor FtsY
gmk	COG0194	Guanylate kinase
hisS	COG0124	Histidine-tRNA ligase
ileS	COG0060	Isoleucine-tRNA ligase 1
infB	COG0532	Translation initiation factor IF-2
infC	COG0290	Translation initiation factor IF-3
ksgA	COG0030	Ribosomal RNA small subunit methyltransferase A
lepA	COG0481	Elongation factor 4
leuS	COG0495	Leucine-tRNA ligase
ligA	COG0272	DNA ligase
nusA	COG0195	Transcription termination/antitermination protein NusA
nusG	COG0250	Transcription termination/antitermination protein NusG
pgk	COG0126	Phosphoglycerate kinase
pheS	COG0016	Phenylalanine-tRNA ligase alpha subunit
pheT	COG0073	Phenylalanine-tRNA ligase beta subunit
prfA	COG0216	Peptide chain release factor 1
pyrG	COG0504	CTP synthase
recA	COG0468	DNA recombination and repair protein
rbfA	COG0858	30S ribosome-binding factor
rnc	COG0571	Ribonuclease 3
rplA	COG0081	50S ribosomal protein L1
rplB	COG0090	50S ribosomal protein L2
rplC	COG0087	50S ribosomal protein L3
rplD	COG0088	50S ribosomal protein L4
rplE	COG0094	50S ribosomal protein L5
rplF	COG0097	50S ribosomal protein L6
rplI	COG0359	50S ribosomal protein L9
rplJ	COG0244	50S ribosomal protein L10
rplK	COG0080	50S ribosomal protein L11

rplL	COG0222	50S ribosomal protein L7/L12
rplM	COG0102	50S ribosomal protein L13
rplN	COG0093	50S ribosomal protein L14
rplO	COG0200	50S ribosomal protein L15
rplP	COG0197	50S ribosomal protein L16
rplQ	COG0203	50S ribosomal protein L17
rplR	COG0256	50S ribosomal protein L18
rplS	COG0335	50S ribosomal protein L19
rplT	COG0292	50S ribosomal protein L20
rplU	COG0261	50S ribosomal protein L21
rplV	COG0091	50S ribosomal protein L22
rplW	COG0089	50S ribosomal protein L23
rplX	COG0198	50S ribosomal protein L24
rpmA	COG0211	50S ribosomal protein L27
rpmC	COG0255	50S ribosomal protein L29
rpmI	COG0291	50S ribosomal protein L35
rpoA	COG0202	DNA-directed RNA polymerase subunit alpha
rpoB	COG0085	DNA-directed RNA polymerase subunit beta
rpoC	COG0086	DNA-directed RNA polymerase subunit beta’
rpsB	COG0052	30S ribosomal protein S2
rpsC	COG0092	30S ribosomal protein S3
rpsD	COG0522	30S ribosomal protein S4
rpsE	COG0098	30S ribosomal protein S5
rpsF	COG0360	30S ribosomal protein S6
rpsG	COG0049	30S ribosomal protein S7
rpsH	COG0096	30S ribosomal protein S8
rpsI	COG0103	30S ribosomal protein S9
rpsJ	COG0051	30S ribosomal protein S10
rpsK	COG0100	30S ribosomal protein S11
rpsL	COG0048	30S ribosomal protein S12
rpsM	COG0099	30S ribosomal protein S13
rpsO	COG0184	30S ribosomal protein S15
rpsP	COG0228	30S ribosomal protein S16
rpsQ	COG0186	30S ribosomal protein S17
rpsR	COG0238	30S ribosomal protein S18
rpsS	COG0185	30S ribosomal protein S19
rpsT	COG0268	30S ribosomal protein S20
secA	COG0653	Protein translocase subunit SecA
secG	COG1314	Protein-export membrane protein SecG
secY	COG0201	Protein translocase subunit SecY
serS	COG0172	Serine-tRNA ligase
smpB	COG0691	SsrA-binding protein
tig	COG0544	Trigger factor
tilS	COG0037	tRNA(Ile)-lysidine synthase

truB	COG0130	tRNA pseudouridine synthase B
tsaD	COG0533	tRNA N6-adenosine threonylcarbamoyltransferase
tsf	COG0264	Elongation factor Ts
uvrB	COG0556	UvrABC system protein B
ybeY	COG0319	Endoribonuclease YbeY
ychF	COG0012	Ribosome-binding ATPase YchF

Table S4. *Streptococcus* genome sequences used in multigene-based phylogenomic treeing approach.

Species	GenBank accession number	Group
Streptococcus porcorum DSM28302[T]	[a]2928214140	NA
Streptococcus parasuis DSM 29126[T]	[a]2928335100	Suis
Streptococcus mitis NCTC 12261[T]	GCA_000148585.3	Mitis
Streptococcus parasanguinis NCTC12854[T]	GCA_000164675.2	Mitis
Streptococcus infantis ATCC 700779[T]	GCA_000187465.1	Mitis
Streptococcus peroris ATCC 700780[T]	GCA_000187585.1	Mitis
Streptococcus macacae NCTC 11558[T]	GCA_000187995.3	Mutans
Streptococcus ictaluri DSM 21677[T]	GCA_000188015.3	Pyogenic
Streptococcus vestibularis ATCC 49124[T]	GCA_000188295.1	Salivarius
Streptococcus constellatus subsp. *pharyngis* SK1060[T]	GCA_000223295.2	Gordonii
Streptococcus ratti FA-1[T]	GCA_000286075.1	Mutans
Streptococcus suis S735[T]	GCA_000294495.1	Suis
Streptococcus oralis subsp. *tigurinus* AZ_3a[T]	GCA_000344275.1	Mitis
Streptococcus minor DSM 17118[T]	GCA_000377005.1	Suis
Streptococcus ovis DSM 16829[T]	GCA_000380125.1	Suis
Streptococcus thoraltensis DSM 12221[T]	GCA_000380145.1	Pluranimalium
Streptococcus oralis subsp. *dentisani* CECT 7747[T]	GCA_000382825.1	Mitis
Streptococcus hyovaginalis DSM 12219[T]	GCA_000420785.1	Pluranimalium
Streptococcus devriesei DSM 19639[T]	GCA_000423725.1	Mutans
Streptococcus castoreus DSM 17536[T]	GCA_000425025.1	Pyogenic
Streptococcus equi subsp. *ruminatorum* CECT 5772[T]	GCA_000706805.1	Pyogenic
Streptococcus sinensis HKU4[T]	GCA_000767835.1	Gordonii
Streptococcus phocae subsp. *salmonis* C-4[T]	GCA_000772915.1	Pyogenic
Streptococcus uberis NCTC3858[T]	GCA_000785845.1	Pyogenic
Streptococcus phocae subsp. *phocae* ATCC 51973[T]	GCA_001302265.1	Pyogenic
Streptococcus pneumoniae DSM 20566[T]	GCA_001457635.1	Mitis

Streptococcus iniae QMA0248^{T}	GCA_001595425.1	Pyogenic
Streptococcus gallolyticus subsp. *gallolyticus* DSM 16831^{T}	GCA_002000985.1	Bovis
Streptococcus pyogenes DSM 20565^{T}	GCA_002055535.1	Pyogenic
Streptococcus pseudopneumoniae ATCC BAA-960^{T}	GCA_002087075.1	Mitis
Streptococcus pluranimalium TH11417	GCA_002953735.1	Pluranimalium
Streptococcus ruminantium GUT187^{T}	GCA_003609975.1	Suis
Streptococcus halichoeri CCUG 48324^{T}	GCA_009870755.1	Pyogenic
Streptococcus oriscaviae HKU75^{T}	GCA_018137985.1	Suis
Lactococcus lactis ATCC 19435^{T}	GCA_900099625.1	Outgroup
Streptococcus equi subsp. *equi* NCTC9682^{T}	GCA_900156215.1	Pyogenic
Streptococcus agalactiae NCTC 8181^{T}	GCA_900458965.1	Pyogenic
Streptococcus acidominimus NCTC 12957^{T}	GCA_900459045.1	Suis
Streptococcus dysgalactiae subsp. *equisimilis* NCTC 13762^{T}	GCA_900459095.1	Pyogenic
Streptococcus constellatus subsp. *constellatus* NCTC 11325^{T}	GCA_900459125.1	Gordonii
Streptococcus downei NCTC 11391^{T}	GCA_900459175.1	Sobrinus
Streptococcus dysgalactiae subsp. *dysgalactiae* NCTC 13731^{T}	GCA_900459225.1	Pyogenic
Streptococcus massiliensis NCTC 13765^{T}	GCA_900459365.1	Gordonii
Streptococcus infantarius NCTC 13760^{T}	GCA_900459445.1	Bovis
Streptococcus equi subsp. *zooepidemicus* NCTC 4676^{T}	GCA_900459475.1	Pyogenic
Streptococcus gallolyticus subsp. *macedonicus* NCTC 13767^{T}	GCA_900459545.1	Bovis
Streptococcus thermophilus NCTC 12958^{T}	GCA_900474985.1	Salivarius
Streptococcus gordonii NCTC 7865^{T}	GCA_900475015.1	Gordonii
Streptococcus ferus DSM 20646^{T}	GCA_900475025.1	Mutans
Streptococcus mutans FDAARGOS 1458^{T}	GCA_900475095.1	Mutans
Streptococcus sobrinus NCTC 12279^{T}	GCA_900475395.1	Sobrinus
Streptococcus porcinus NCTC 10999^{T}	GCA_900475415.1	Pyogenic
Streptococcus cristatus NCTC 12479^{T}	GCA_900475445.1	Gordonii
Streptococcus sanguinis NCTC 7863^{T}	GCA_900475505.1	Gordonii

Streptococcus lutetiensis NCTC 13774^T	GCA_900475675.1	Bovis
Streptococcus intermedius NCTC 11324^T	GCA_900475975.1	Gordonii
Streptococcus australis NCTC 13166^T	GCA_900476055.1	Mitis
Streptococcus gallolyticus subsp. *pasteurianus* NCTC13784^T	GCA_900478025.1	Bovis
Streptococcus salivarius NCTC 8618^T	GCA_900636435.1	Salivarius
Streptococcus anginosus subsp. *anginosus* NCTC 10713^T	GCA_900636475.1	Gordonii
Streptococcus urinalis NCTC 13766^T	GCA_900636885.1	Pyogenic
Streptococcus oralis subsp. *oralis* NCTC 11427^T	GCA_900637025.1	Mitis
Streptococcus pseudoporcinus NCTC 13786^T	GCA_900637075.1	Pyogenic

[a]Genomes downloaded from JGI (2).

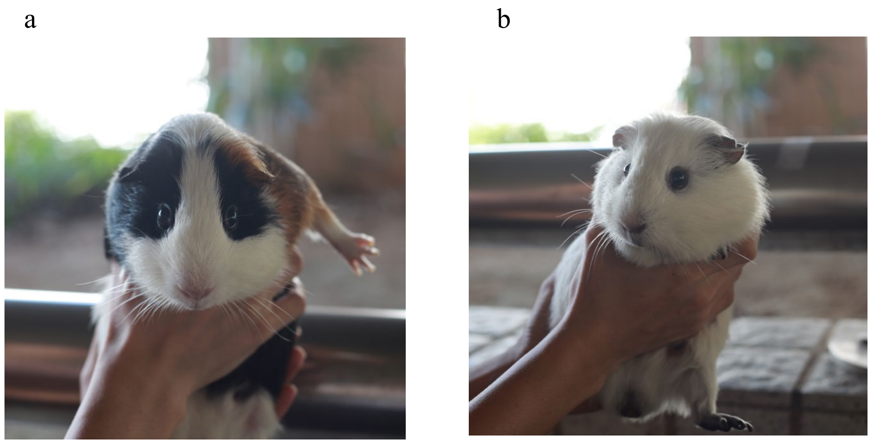

Fig. S1. The two guinea pigs purchased from a local pet store; (a) GP1 and (b) GP2.

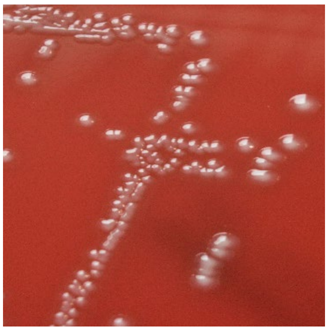

Fig. S2. Colony morphology of HKU75^{T}. Growth on sheep blood agar after 24 h of incubation at 37°C with 5% CO_2.

References

1. Davis JJ, Wattam AR, Aziz RK, Brettin T, Butler R, Butler RM, Chlenski P, Conrad N, Dickerman A, Dietrich EM, Gabbard JL, Gerdes S, Guard A, Kenyon RW, MacHi D, Mao C, Murphy-Olson D, Nguyen M, Nordberg EK, Olsen GJ, Olson RD, Overbeek JC, Overbeek R, Parrello B, Pusch GD, Shukla M, Thomas C, Vanoeffelen M, Vonstein V, Warren AS, Xia F, Xie D, Yoo H, Stevens R. 2020. The PATRIC Bioinformatics Resource Center: expanding data and analysis capabilities. *Nucleic acids research* 48:D606-D612.
2. Nordberg H, Cantor M, Dusheyko S, Hua S, Poliakov A, Shabalov I, Smirnova T, Grigoriev IV, Dubchak I. 2014. The genome portal of the Department of Energy Joint Genome Institute: 2014 updates. *Nucleic Acids Res* 42:D26-31.
